# Systematic alveolar recruitment after cardiac surgery

**DOI:** 10.1186/cc14351

**Published:** 2015-03-16

**Authors:** AL Lafarge, CK Kerneis, F Scalbert, LL Larnier, AB Brusset, PE Estagnasie, PS Squara

**Affiliations:** 1 Clinique Ambroise Paré, Neuilly-sur-Seine, France

## Introduction

We designed a pilot study to evaluate the interest of an early systematic acute recruitment maneuver (ARM) in postcardiac surgery hypoxemic patients in order to properly design a larger trial.

## Methods

This randomized controlled trial included consecutive patients operated on in our institution. Three hours after surgery, hypoxemic patients (PaO_2_ <300 mmHg, FIO_2 _= 1) were randomly assigned to ARM or control (H0). ARM was performed by applying once a positive end-expiratory pressure of 35 cmH_2_O during 45 seconds. Blood gases and hemodynamic variables were collected at H1, H8, H24 and H48. The primary endpoint was the duration of mechanical ventilation (MV). Secondary endpoints were survival rate, ICU length of stay and the occurrence of pneumonia.

## Results

**Figure 1:**
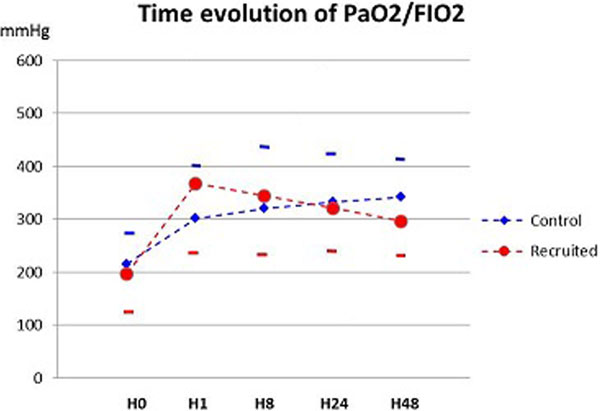


We included 124 patients, age 67.5 ± 10.6 years, M/F sex ratio 95/29, left ventricle ejection fraction 58.8 ± 10.6%, forced expiratory volume 94 ± 23% of the predicted value, bypass/valve ratio 82/53. The preoperative and postoperative PaO_2_/FIO_2_ were 401 ± 66 and 204 ± 66 mmHg, respectively (*P *< 0.0001). The hemodynamic and ventilation status as well as the fluid and inotrope supports were comparable in the two groups. At H1, PaO_2_/FIO_2_ was 367 ± 15 in the recruited group versus 299 ± 15 mmHg in the control group, *P *= 0.002. At H8 and 24 the difference was not significant. At H48, the PaO_2_/FIO_2_ was lower in the recruited group (296 ± 10 vs. 343 ± 11 mmHg, *P *= 0.003) (Figure 1). The duration of mechanical ventilation (invasive + non-invasive) was lower in the recruited group (total 6.4 ± 1.4 vs. 8.4 ± 1.4 hours, *P *= 0.02). The survival rate, the length of stay in the ICU and the occurrence of pneumonia were similar in the two groups (*P >*0.2).

## Conclusion

We can speculate that the inverse evolution of the blood oxygenation between the ARM group versus control may be due to: barotraumatism of normal alveoli during the ARM and/or a higher de-recruitment rate after ARM due to the shorter mechanical ventilation support. This pilot study shows that a unique ARM decreased the duration of MV in cardiac surgery patients but this may have subsequent detrimental effects on blood oxygenation.

